# Mechanical and Fracture Properties of Fly Ash Geopolymer Concrete Addictive with Calcium Aluminate Cement

**DOI:** 10.3390/ma12182982

**Published:** 2019-09-15

**Authors:** Yamin Wang, Shaowei Hu, Zhen He

**Affiliations:** 1 School of Water Resources and Hydropower Engineering, Wuhan University, Wuhan 430072, China; wangyam@whu.edu.cn (Y.W.); hezhen@whu.edu.cn (Z.H.); 2 Department of Materials and Structural Engineering, Nanjing Hydraulic Research Institute, Nanjing 210024, China

**Keywords:** fly ash geopolymer concrete, calcium aluminate cement, mechanical properties, fracture properties

## Abstract

In this paper, the mechanical and fracture properties of fly ash geopolymer concrete (FAGC) mixed with calcium aluminate cement (CAC) were explored. Fly ash was partially replaced by CAC with 2.5%, 5% and 7.5%. The results exhibit that the mechanical and fracture behaviors of FAGC are significantly influenced by CAC content. Based on the formation of more aluminum-rich gels, C-(A)-S-H and C-S-H gels, with the increase of CAC content, the compressive strength, splitting tensile strength and elastic modulus improved. Meanwhile, the peak load and effective fracture toughness show a monotone increasing trend. In addition, because C-S-H gels absorbed more energy, the fracture energy of FAGC increases. The maximal peak load, double-K fracture toughness and fracture energy reached up to1.79 kN, 4.27 MPam^0.5^, 10.1 MPam^0.5^ and 85.8 N/m with CAC content of 7.5%, respectively.

## 1. Introduction

Globally, concrete is the most widely used building material; However, the production of one ton of cement will emit 600–800 kg of CO_2_, which accelerates global warming [1]. It is necessary for concrete to switch over from Portland cement to a greener and environmentally friendly alternative binder with desirable mechanical and durability properties [2]. As one of the novel types of aluminosilicate inorganic polymer materials, geopolymer is produced by the reaction of solid aluminosilicate source materials and high concentration of alkali activator, which was described by Davidovits in the 1970s [3]. Through a large number of studies, fly ash geopolymer was reported to have many superior mechanical performances, such as high compressive strength, negligible shrinkage, good resistance to acid, and thermal stability, etc. [4,5,6,7,8]. In order to expand the application range of fly ash geopolymer, a large number of studies have been conducted to improve the performance of geopolymer by adjusting the chemical composition of the aluminosilicate raw materials. The preferred methods are to incorporate calcium-rich or silica-rich source materials, such as Portland cement, blast furnace slag, silica fume, rice husk ash, metakaolin, and nano-particles etc. [9,10,11,12].

In fact, not only silicon-rich materials and calcium-rich materials can improve the properties of geopolymers, but also aluminum-rich materials can achieve this goal. For alkali activator materials, Criado et al. [13] claimed that the amount of $Al{\left(OH\right)}_{4}{}^{-}$ tetrahedral groups significantly affected the number of aluminosilicate gels, owing to $Al{\left(OH\right)}_{4}{}^{-}$ tetrahedral groups can attract positive charges. Therefore, under the environment of alkali activator, increasing the content of reactive aluminum can increase the content of $Al{\left(OH\right)}_{4}{}^{-}$ in geopolymer mixes. As a kind of special cement, calcium aluminate cement (CAC) consists of various aluminates with the content ranging from 40% to 90% [14]. CAC has many excellent properties, such as high early strength, high temperature resistance and wide corrosion resistance [15,16]. The main components of CAC contain CaO·Al_2_O_3_ (CA), CaO·2Al_2_O_3_ (CA_2_) and a portion of 12CaO·7Al_2_O_3_ (C_12_A_7_) [17].

The hydration products vary with curing temperature and humidity. Under low temperature (<20 °C) conditions, the main hydration product is CaO·Al_2_O_3_·10H_2_O (CAH_10_) [18], once the temperature is greater than 20 °C, the main hydration products are 2CaO·Al_2_O_3_·8H_2_O (C_2_AH_8_) and Al(OH)_3_ (AH_3_) [19]. According to the literature [20], when the temperature and humidity are increased, CAH_10_ and C_2_AH_8_, which are metastable phases, will transform into stable 3CaO·Al_2_O_3_·6H_2_O (C_3_AH_6_) and AH_3_. In an alkaline environment, AH_3_ will react with $OH^{-}$ to form $Al{\left(OH\right)}_{4}{}^{-}$ [21]. Building on these results, the CAC can be used as an aluminum-rich material to improve the properties of fly ash geopolymer.

Very few studies have investigated the effect of CAC as the aluminum-rich material on the alkali activator materials. Arbi et al. [22] investigated the blast furnace slag and natural rock mixed with CAC. The results showed that the former system obtained C-(A)-S-H gel and sulfoaluminate in the medium alkaline medium. Vafaei and Allahverdi [14] studied the influence of CAC on natural pozzolan. They found that CAC increased the aluminum content of the binder and promoted the geopolymerization to form more aluminosilicate gels. Reig et al. [23] explored the effect of red clay brick waste with CAC. The results identified that CAC accelerated the activation process of red bricks and the compressive strength achieved 50 MPa. Tao et al. [24] explored the mechanical properties of fly ash geopolymer concrete (FAGC) with CAC. The results identified that the optimal replacement rate of CAC is 10% on the compressive strength of 7 days and 28 days. So far, fly ash geopolymer concrete as the most widely explored alkali-activated material, there are not enough studies on the properties of FAGC combined with CAC under the high temperature curing condition.

The combination of FAGC and CAC has been, for obvious reasons, of particular interest. The CAC, which will refer to as the aluminum-rich material, contains reactive aluminum and calcium ions, both of which can be used to improve the properties of FAGC. As a kind of new composite material, appropriate researches on the basic properties of FAGC are required to provide support for the engineering applications, among which mechanical properties and the fracture characteristics are particularly important. 

Above all, the aim of this study is to explore the influence of CAC content on the mechanical and fracture properties of FAGC cured at 75 °C, including a portion of CAC replacement percentage (2.5%–7.5%). For mechanical properties, the compressive strength, splitting tensile strength and elastic modulus were selected for testing. In addition, for fracture properties, by means of three-point bending test, the load-crack mouth opening displacement curve, the peak load, the fracture energy and the double-K fracture toughness were obtained.

## 2. Materials and Methods

### 2.1. Material

**Figure 1:**
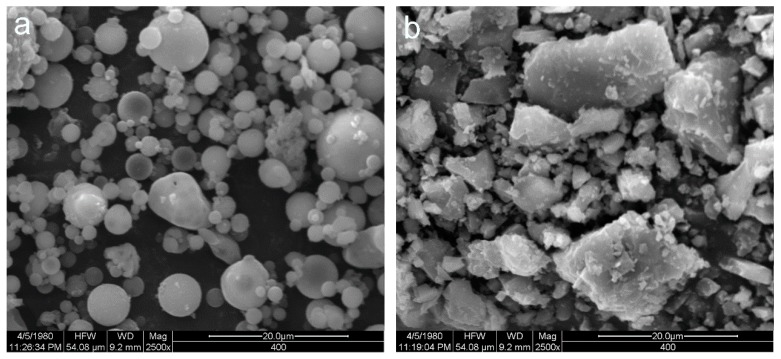
SEM images of (**a**) fly ash and (**b**) calcium aluminate cement (CAC) particles.

In this study, the fly ash obtained from the Ningdong power plant in China were used. CAC purchased in Jianai Special Aluminates Co., Ltd. (Zhengzhou, China). Fly ash and CAC were analyzed by XRF to determine their chemical compositions, which are presented in Table 1. The main compositions of fly ash are SiO_2_ and Al_2_O_3_, and the main compositions of CAC are Al_2_O_3_ and CaO. The morphology of fly ash and CAC were observed by scanning electron microscope (SEM) (Quanta, Philips corporation, Eindhoven, Holland), as shown in Figure 1. The alkali activator consists of sodium silicate (Na_2_SiO_3_) (composed of 25.89% SiO_2_ and 8.11% Na_2_O by mass), with a SiO_2_/Na_2_O molar ratio of 3.3, as well as sodium hydroxide (NaOH) pellets (96%). The alkaline activator is prepared by mixing sodium hydroxide and sodium silicate [25]. River sand and gravel were used as fine aggregate and coarse aggregate in accordance with the Chinese standard JGJ 52-2006 [26]. For river sand, the density was 2645 kg/m^3^, the sand was 2645 kg/m^3^, absorption was 2.9%, and fineness modulus was 2.63, respectively. For gravel, the bulk density was 2530 kg/m^3^, and water absorption was 1.83%, respectively [27].

### 2.2. Specimen Preparation

The mixtures were mixed in a laboratory mixer. To produce alkali activator, NaOH was dissolved in distilled water and stirred uniformly, and then mixed with Na_2_SiO_3_ solution. Firstly, gravel, river sand and fly ash was poured into a laboratory mixer, and stirred for 4 min. After the dry mixing, the alkaline activator, extra water and sodium gluconate (sg) were then added in the mix gradually, and the wet mixed for 2 min. The fresh concrete mixture was cast in the molds of cubes and beams, without any compaction to fill spaces of molds by its own weight. The molds were then stored in a curing box at the temperature of 75 °C for 16 h. Before testing, the samples were removed from molds after curing and left in the room with the temperature varying between 18 and 23 °C. The mixes description and the proportions of ingredients are as per Table 2.

### 2.3. Compressive Strength, Splitting Tensile Strength and Elastic Modulus

To investigate the influence of CAC content on the mechanical properties of FAGC, the compressive strength, splitting tensile strength and elastic modulus were determined. The test specimen size and test procedure were carried out in accordance with Chinese standard GB/T 50081-2002 [28]. The 100 × 100 × 100 mm^3^ cube specimen was selected for the compressive strength test, the 150 × 150 × 150 mm^3^ prism specimen was used for the splitting tensile strength test, and the 150 × 150 × 300 mm^3^ prism specimen was used for the elastic modulus test. The loading rates of compressive strength and splitting tensile strength were set to 2.4 kN/s and 50 N/s, respectively.

### 2.4. Three Point Bending Test

**Figure 2:**
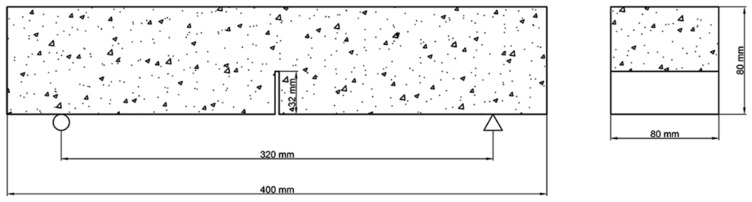
Schematic diagram of the three-point bending test.

To determine fracture properties of the FAGC specimens, the three-point bending test was conducted reference to the RILEM guidelines [29,30]. To explore the fracture behaviors of FAGC, the size of the notched beam used in three-point bending test is 80 mm × 80 mm × 400 mm. In the forming process of the beam, the notch is made of steel plate. The parameters of the precast crack are set as follows: The thickness is 3 mm, depth is 32 mm, and the tip angle is 15°. Four groups of specimens contain sixteen specimens. The beam is placed on the ball bearing support in the form of notched face down, with a span of 320 mm (Figure 2). The test was carried out on a HUALONG 200C electronic universal testing machine (Shanghai, China) with a load capacity of 20 tons. During the three-point bending test, the loading rate is set to 0.5 mm/min, and a clip gauge was installed at the mouth of the notch to record the data of crack mouth opening displacement (CMOD). 

## 3. Results and Discussion

### 3.1. Compressive Strength

**Figure 3:**
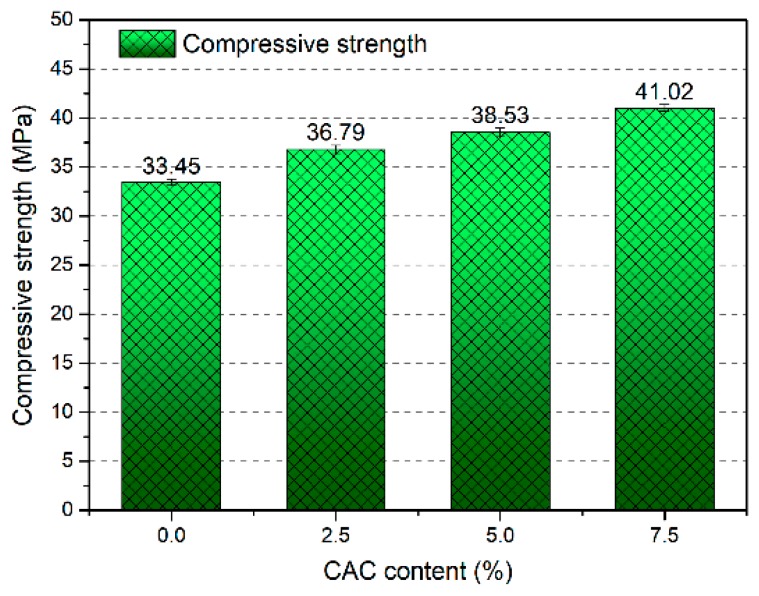
The compressive strength of fly ash geopolymer concrete (FAGC) with different CAC content.

Figure 3 and Table 3 express the effect of CAC content on compressive strength developments of FAGC. Obviously, the CAC content plays a major role in the compressive strength of FAGC. It can be seen from Figure 3 that the compressive strength of FAGC improves from 33.45 MPa to 41.02 MPa, when the CAC content grows from 0 to 7.5%. In addition, the value of compressive strength is 36.79 MPa with 2.5% and 38.53 MPa with 5%, respectively. The value of compressive strength with CAC content of 2.5%, 5% and 7.5% is 9.99%, 15.19% and 22.63% higher than that with plain geopolymer concrete, respectively. Taking into account the CAC content, increasing CAC content always resulted in the enhancement of compressive strength. Based on previous literature [31,32], the polycondensation reaction of geopolymer will produce much more Si-O-Al bonds, which significantly affect the development of compressive strength. The aluminum plays an important role in the polycondensation of geopolymer; what is more, the geopolymer mechanical properties are affected by the calcium content. As a good source of reactive aluminum and additional calcium, the CAC can be taken up into the geopolymerization process, the high amount of aluminum promotes the formation of more aluminum-rich gels, the additional calcium favors the formation of aluminum-substitute calcium silicate hydrate (C-(A)-S-H) and C-S-H gels [30], which may further promote the properties of geopolymers. In summary, the addition of CAC has positive influence on the strength development of FAGC.

**Figure 4:**
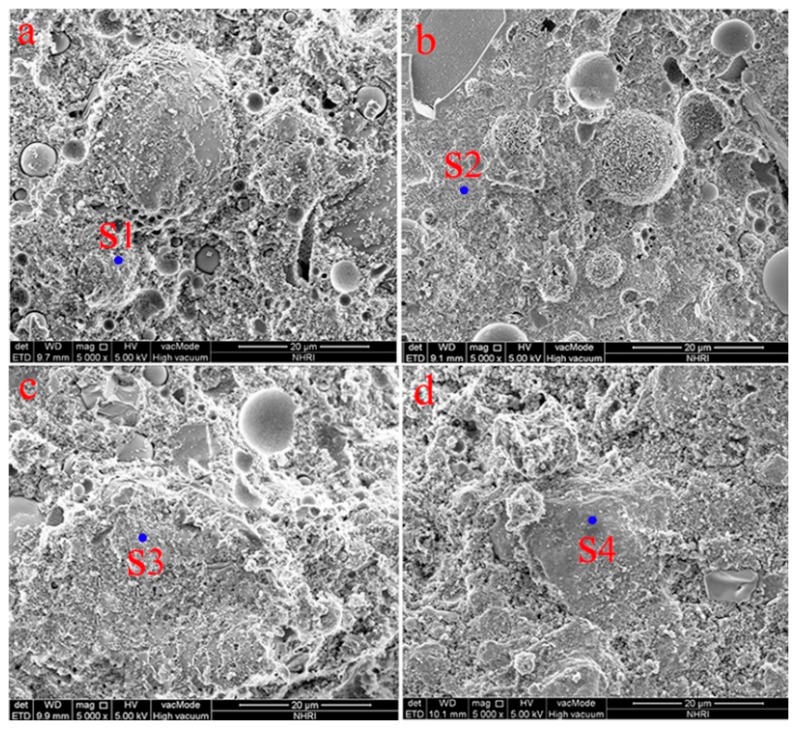
SEM Images of FAGC: (**a**) CAC-0; (**b**) CAC-2.5%; (**c**) CAC-5%; (**d**) CAC-7.5%.

To observe the microstructure of FAGC with different CAC contents, SEM and EDS analysis were performed. The results are shown in Figure 4 and Table 4. In Figure 4a, for FAGC with 0% CAC, there are many unreacted FA particles, what is more, the improper bonding of FA particles with binders can be observed, which result in the generation of weak points and decrease compressive strength [33]. With the increase of CAC content, the unreacted FA particles to be less, the aluminosilicate gels to be more compact and the amount of C-A-S-H gel increase, as shown in Figure 4b–d. Based on the results of EDS, the presence of calcium, sodium, silicon and aluminum confirm the C-A-S-H gel in coexistence with N-A-S-H gel. It can also be noticed that Ca/Na increased and Si/Al ratio decreased with an increase in the CAC content from 0% to 7.5%, which favor the amount of C-A-S-H gel increase. 

### 3.2. Splitting Tensile Strength

**Figure 5:**
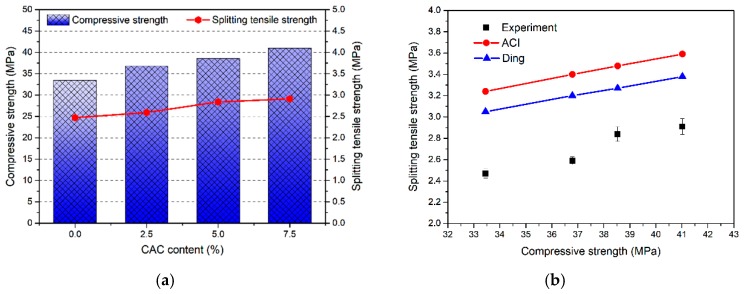
(**a**) Relationship between splitting tensile strength and compressive strength; (**b**) relationship of splitting tensile strength between experiment and proposed equations.

As summarized in Figure 5a and Table 3, it can be seen that the CAC has a significant effect on the splitting tensile strength of FAGC. With the increase of CAC content, the splitting tensile strength of FAGC improved. Typically, for the different CAC contents, splitting tensile strength is 2.47 MPa with plain geopolymer concrete, 2.59 MPa with 2.5%, 2.84 MPa with 5%, and 2.91 MPa with 7.5%. The value of splitting tensile strength with CAC content of 2.5%, 5% and 7.5% is 4.86%, 14.98% and 17.81% higher than that with plain geopolymer concrete, respectively.

Reference to the literature [34], some recommended equations and empirical equations can be used to predict the splitting tensile strength from compressive strength of FAGC. The empirical equations recommended in codes of practice were used to predict splitting tensile strength. For instance, American concrete institute (ACI) Building Code 318 [35] and Ding et al. [34] can be used to predict the splitting tensile strength, which is expressed by Equations (1) and (2).

(1) $$f_{t}=0.56\sqrt{f_{c}}$$

where $f_{t}$ is splitting tensile strength (MPa) and $f_{c}$ is compressive strength (MPa),

(2) $$f_{t}=0.527\sqrt{f_{c}}$$

The splitting tensile strength of FAGC with different CAC contents obtained from tests, and those obtained by Equations (1) and (2) are expressed in Figure 5b. It is clear that the splitting tensile strength of FAGC is overestimated by ACI 318 Model and the equation proposed by Ding et al. [34]. For the given compressive strength of 41.02 MPa, the splitting tensile strength of FAGC is 2.91 MPa. The results obtained by Equations (1) and (2) are 3.59 MPa and 3.38 MPa, respectively, which are 23.37% and 16.15% higher compared with the experimental result, respectively.

### 3.3. Elastic Modulus

**Figure 6:**
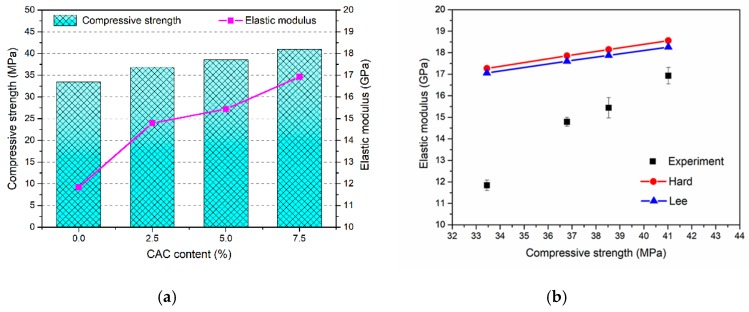
(**a**) Relationship between elastic modulus and compressive strength; (**b**) relationship of elastic modulus between experiment and proposed equations.

As one of the important mechanical properties of concrete, the value of elastic modulus varies with the compressive strength. The mean value of elastic modulus for FAGC with different CAC contents were obtained from tests, and the Equations are shown in Figure 6a and Table 3. What is more, Figure 6b shows the variation of elastic modulus with respect to compressive strength. Elastic modulus increases with the improvement of compressive strength. The elastic modulus of FAGC without CAC is 11.84 GPa corresponding to the compressive strength of 33.45 MPa. With the increasing of CAC content, an increase in the elastic modulus of FAGC was observed. The values of elastic modulus are up to 14.79 GPa and 15.44 GPa with CAC content of 2.5% and 5% reference to 36.79 MPa and 38.53 MPa, respectively. The highest elastic modulus achieves up to 16.93 GPa with the CAC content of 7.5% for compressive strength of 41.02 MPa. Relative to the FAGC without CAC, the improvements of elastic modulus of FAGC with 2.5%, 5% and 7.5% reach up to 24.92%, 30.41% and 42.99%, respectively. These results can be attributed to the Young’s modulus of C-S-H gel which is equal to around 16–44 GPa, the Young’s modulus of N-A-S-H gel is about 17–18 GPa [36,37,38], which is significantly lower than that of C-S-H gel. Therefore, the increasing of C-S-H content resulted in the improvement of elastic modulus with different CAC contents.

Generally, the elastic modulus of concrete is believed to be related to compressive strength. Thus, some empirical equations can be used to predict the elastic modulus from compressive strength. The Equations (3) and (4) proposed by Hardjito et al. [39] and Lee and Lee [40] based on test results of geopolymer concrete.

(3) $$E=2070\sqrt{f_{c}}+5300,$$

where E is the elastic modulus (GPa),

(4) $$E=5300\sqrt[{3}]{f_{c}}$$

The comparison between the elastic modulus obtained by experiment and the predicted by the above equations are plotted in Figure 6b. The experimental values are lower than those calculated reference to empirical equations of Equations (3) and (4).

### 3.4. Fracture Properties

#### 3.4.1. Load-CMOD Curves

**Figure 7:**
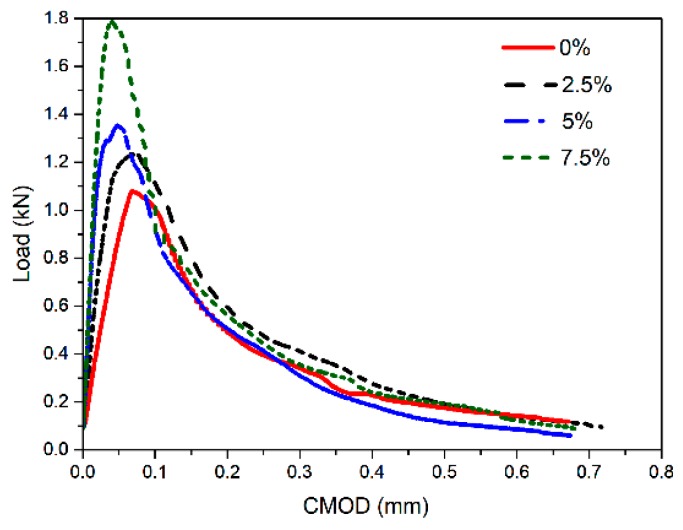
Load- crack mouth opening displacement (CMOD) curves of FAGC with different CAC contents.

The fracture behavior of concrete can be expressed by means of the complete load-CMOD curve. The typical load-CMOD curves of FAGC exposed to four different CAC contents are shown in Figure 7. Based on these curves, it can be seen that FAGC is almost in a state of linear elastic deformation at the start of the loading. Before the load reached up to the initial load, there is not an obvious observation on nonlinear deformation. For the ascending branches of the load-CMOD curve, the slope increase with the increasing of CAC content, owing to the compressive strength and elastic modulus of FAGC increased with the improvement of CAC content. In addition, the crack initiated at the moment of reaching the peak load, then the peak curve shows a decreasing trend. Like the ascending branch, the descending branch can also express the fracture property of the cracked specimen, which is the ductility of FAGC. With the increase of the compressive strength of FAGC, the slope presents a tendency to decrease. This shows that the ductility of FAGC reduced when CAC content increased. Before complete failure, the FAGC with CAC of 7.5%, which has the highest strength, showed greater stretch of the descending branch.

#### 3.4.2. Peak Load

**Figure 8:**
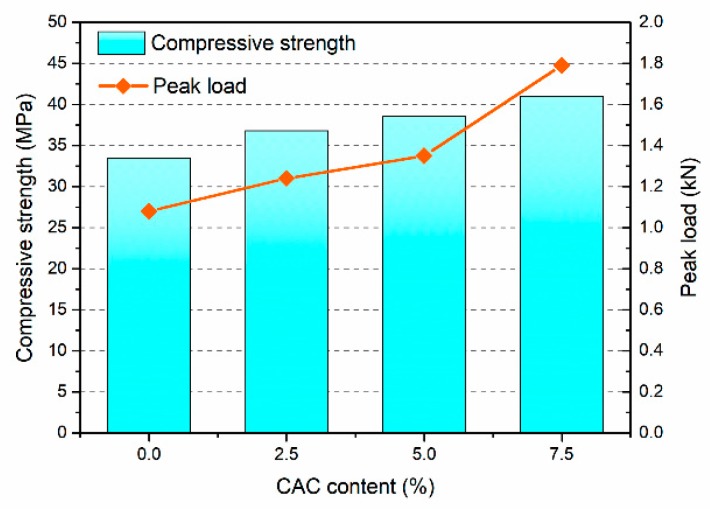
Peak loads of FAGC with different CAC contents.

Table 5 presents the peak loads of FAGC with different CAC contents. The corresponding relationship between peak load and compressive strength of FAGC are presented in Figure 8. The peak load increases with the increase of compressive strength. The peak load of FAGC varies in the range from 1.08 to 1.79 kN. The FAGC mixed with 7.5% CAC with the maximal peak load and compressive strength. Obviously, when the CAC content grows up to 2.5%, 5% and 7.5%, the peak loads of FAGC are 1.24 kN, 1.35 kN and 1.79 kN, which are 14.81%, 25% and 65.74% larger than that of FAGC without CAC, respectively. The CAC content greatly influences the compressive strength, which leads to the variation of peak load at the same time. As expected, the peak loads of FAGC increase with the increase of CAC content, that is to say, improve with compressive strength.

#### 3.4.3. Fracture Energy

Fracture energy refers to the energy needed to generate cracks per unit area. As shown in Equation (5), it is a method to obtain the fracture energy of the three-point bending specimen by calculating the area surrounded by the measured load-CMOD curve divided by the ligament area. In order to simplify the test, when the measured crack work is close to the actual fracture energy, the end point is close to the point of complete failure. According to the literature [30], the constant value of the far tail can be used to calculate the true fracture energy. In this study, the test stops when the descending branch of the load-CMOD curve is full-tailed. Moreover, in the calculation of fracture energy, the self-weight of the sample is not taken into account owing to the size of the specimen and the supporting form of the specimen.

(5) $$G_{f}=\frac{W_{0}}{A_{lig}}$$

where $W_{0}$ is equal to the area of load-CMOD curve and $A_{lig}$ is the ligament area (m^2^).

**Figure 9:**
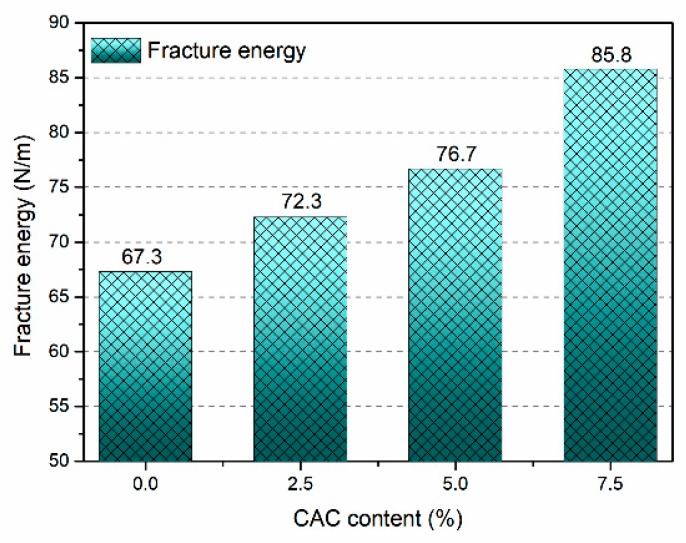
Fracture energy of FAGC with different CAC contents.

In Figure 9, the fracture energy of FAGC is distinctly affected by CAC content. The change in the fracture energy of FAGC has the same trend as that in CAC content. At the beginning, the value of $G_{f}$ of FAGC without CAC is 67.3 N/m; however, when the CAC content grows from 0 to 2.5% and 5%, the $G_{f}$ rapidly increases up to the value of 72.3 N/m and 76.7 N/m. On prolonging the CAC content up to 7.5%, a similar phenomenon, $G_{f}$ tends to increase at a homologous rate, has been observed. The value of $G_{f}$ eventually reaches up to 85.8 N/m. Summarized the above results, it can be concluded that the fracture energy continuously increased with the increasing of CAC content. Consequently, when the CAC content grows up to 2.5%, 5% and 7.5%, the $G_{f}$ of FAGC are 7.4%, 13.91% and 27.49% higher than that of FAGC without CAC, respectively. The reason why increasing CAC content increases the $G_{f}$ is that the CAC provided more calcium and additional reactive aluminum to participate in geopolymerization, which resulted in the Ca/Si to be larger and yield to more C-A-S-H and C-S-H gels. The C-A-S-H and C-S-H gels with more initial micro-cracks resulted in more ductility matrix to absorb more energy during crack propagation. Hence, the increasing of CAC content gives rise to the increase in the $G_{f}$.

#### 3.4.4. Effective Fracture Toughness

Reference to the double-K fracture model [41], the $P_{ini}$ and $a_{0}$, which are initial cracking load and initial notch depth, can be substituted into Equation (6) to calculated the initial cracking fracture toughness $K_{Ic}^{ini}$.

(6) $$K_{Ic}^{ini}=\frac{3P_{ini}S}{2D^{2}B}\sqrt{a_{0}}F_{2}\left(\frac{a_{0}}{D}\right),\;V_{2}\left(\frac{a}{D}\right)=0.76-2.28V_{2}\left(\frac{a}{D}\right)+3.87V_{2}{\left(\frac{a}{D}\right)}^{2}-2.04{\left(\frac{a}{D}\right)}^{3}+\frac{0.66}{{\left(1-\frac{a}{D}\right)}^{2}},$$

where $P_{ini}$ is the initial cracking load (kN), S is the span of TPB beam (mm), D and B is the height and thickness of TPB beam (mm), a and $a_{0}$ is the effective crack length and initial crack length (mm).

Based on the parameters of peak load $P_{max}$ and critical notch length $a_{c}$ for $P_{ini}$ and $a_{0}$, the unstable fracture toughness $K_{Ic}^{un}$ can be calculated by the above formulas with the substituting method. The $P_{ini}$ was obtained by the initial point of non-linearity in the P-CMOD curve [42]. These parameters mentioned above are summarized in Table 5. Based on Tada et al. [43], for the TPB specimen of S/D = 4, the relation between load and CMOD expresses as follows:

(7) $$CMOD=\frac{24Pa}{BDE}V_{2}\left(\alpha \right),\;V_{2}\left(\alpha \right)=0.76-2.28V_{2}\left(\alpha \right)+3.87V_{2}{\left(\alpha \right)}^{2}-2.04{\left(\alpha \right)}^{3}+\frac{0.66}{{\left(1-\alpha \right)}^{2}},$$

where α = ($a+h_{0}/D+h_{0}$); $h_{0}$ is equal to 3 mm; $a_{c}$ can be determined by Equation (7) when P reached up to $P_{max}$ and CMOD is replaced by $CMOD_{c}$; and *E* can be obtained by Equation (8).

(8) $$E=\frac{24Pa_{0}}{C_{i}BD}V_{2}\left(\alpha_{0}\right),$$

in which, $\alpha_{0}$ = ($a_{0}+h_{0}/D+h_{0}$); $C_{i}$ = $CMOD_{i}$/$P_{i}$.

**Figure 10:**
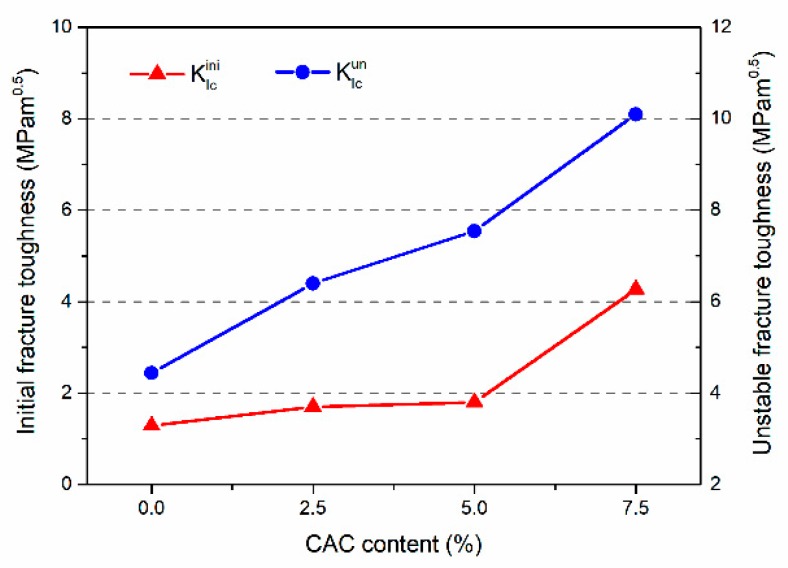
Fracture toughness of FAGC with different CAC contents.

The effective fracture toughness of FAGC with different CAC contents is shown in Figure 10. As shown in Figure 10, it can be seen that $K_{Ic}$ is a monotonically increasing function of the CAC content, and the similar development trend of $G_{f}$ with different CAC contents. For example, when the CAC content of 0%, the $K_{Ic}^{ini}$ is 1.06 MPam^0.5^; however, once the content increases from 0 to 2.5%, 5% and 7.5%, the values of $K_{Ic}^{ini}$ improves up to 1.70, 1.79 and 4.27 MPam^0.5^, respectively. The value of $K_{Ic}^{ini}$ with CAC content of 2.5%, 5% and 7.5% is 60.38%, 68.87% and 302.83% larger than that with plain geopolymer concrete. Afterwards, there is a positive correction between the CAC content and $K_{Ic}^{un}$. Typically, for the different CAC contents, $K_{Ic}^{un}$ is 4.44 MPam^0.5^ with plain geopolymer concrete, 6.4 MPam^0.5^ with 2.5%, 7.54 MPam^0.5^ with 5%, and 10.1 MPam^0.5^ with 7.5%. The values of $K_{Ic}^{un}$ with CAC content of 2.5%, 5% and 7.5% are 44.14%, 69.82% and 127.48%, higher than that with plain geopolymer concrete. These results are owing to that geopolymerization process not only produced the aluminum-rich gels, such as N-A-S-H gels, but also yield C-A-S-H and C-S-H gels, when the FAGC added CAC. According to these results, the effect of CAC on $K_{Ic}$ is getting more and more. Therefore, we can again confidently conclude that CAC content is a decisive factor in determining the properties of FAGC.

## 4. Conclusions

In this study, the effect of CAC content on the mechanical and fracture properties of fly ash geopolymer concrete was investigated. The CAC content ranges from 0 to 7.5%. The compressive strength, splitting tensile strength and elastic modulus of FAGC with different CAC contents were measured. The key fracture parameters, such as load-CMOD curves, peak load, $G_{f}$, $K_{Ic}^{ini}$ and $K_{Ic}^{un}$, were also evaluated from the experimental data for different CAC contents. The following conclusions can be drawn:

(1) The compressive strength, splitting tensile strength and elastic modulus of FAGC improved with the increase of CAC content, the maximal values of them are 41.02 MPa, 2.91 MPa and 16.93 GPa with the growth of 22.63%, 17.81% and 42.99%, respectively, which are corresponding to the CAC content was equal to 7.5%.
(2) The effect of CAC content on $G_{f}$ was significant, and it caused a favorable effect on $G_{f}$. When the CAC content was equal to 7.5%, the maximal value of $G_{f}$ is almost 85.8 N/m, which is owing to the initial micro-crack in the C-S-H gels absorbed much more energy.
(3) The value of $K_{Ic}$ became higher with increasing of CAC content. Typically, for CAC content of 7.5%, the $K_{Ic}^{ini}$ of FAGC reaches up to a maximal value of 4.27 MPam^0.5^. Meanwhile, the biggest value of $K_{Ic}^{un}$ achieves up to 10.1 MPam^0.5^.

## Figures and Tables

**Table 1:** Chemical composition of materials.

Materials	SiO_2_	Al_2_O_3_	Fe_2_O_3_	CaO	MgO	K_2_O	Na_2_O	SO_3_
FA	49.37	32.14	5.20	4.77	1.80	1.59	1.30	1.03
CAC	7.09	49.67	1.99	36.69	0.367	0.56	0.12	0.69

**Table 2:** Details of mix proportion (kg/m^3^).

Mix	Fly Ash	NaOH	Na_2_SiO_3_	Fine Aggregate	Coarse Aggregate	CAC	Water	sg
M0	563.54	44.51	124.55	732.60	599.40	0	132.43	8.45
M1	549.45	44.51	124.55	732.60	599.40	14.09	132.43	8.45
M2	524.09	44.51	124.55	732.60	599.40	28.18	132.43	8.45
M3	521.27	44.51	124.55	732.60	599.40	42.26	132.43	8.45

**Table 3:** Results of mechanical properties of FAGC.

Mix	CAC Content (%)	Compressive Strength (MPa)	Splitting Tensile Strength (MPa)	Elastic Modulus (GPa)
M-0	0	33.45	2.47	11.84
M-1	2.5	36.79	2.59	14.79
M-2	5	38.53	2.84	15.44
M-3	7.5	41.02	2.91	16.93

**Table 4:** Results of EDS of FAGC (atom percent %).

Spectrum	Ca	Na	Al	Si	O	Ca/Na	Si/Al
S1	0.32	4.69	7.64	14.35	60.08	0.07	1.88
S2	1.07	4.08	9.05	14.75	61.71.	0.26	1.62
S3	1.42	3.47	13.51	15.44	60.48	0.41	1.14
S4	2.08	3.42	11.37	11.34	60.49	0.61	1.00

**Table 5:** Experimental results of fracture parameters.

Mix	CAC Content (%)	$P_{ini}\;(N)$	$P_{max}\;(N)$	$CMOD_{c}\;(\mathrm{mm})$	$a_{c}\;(\mathrm{mm})$
M-0	0	621.1	1079	0.0695	36.5
M-1	2.5	817.6	1236	0.06824	42.3
M-2	5	862.4	1353.6	0.04853	44.1
M-3	7.5	1259.6	1788.4	0.0377	44.4
